# Precise chronology of hydrological changes at ∼4.2 kyr in Central China to assess the impact of flooding on Neolithic societies

**DOI:** 10.1093/nsr/nwaf567

**Published:** 2025-12-11

**Authors:** Jin Liao, Christopher C Day, Chaoyong Hu, Yuhui Liu, Gideon M Henderson

**Affiliations:** State Key Laboratory of Geomicrobiology and Environmental Changes, China University of Geosciences, Wuhan 430074, China; Department of Hydrogeology, Wuhan Center, China Geological Survey, Wuhan 430205, China; Department of Earth Sciences, University of Oxford, Oxford OX1 3AN, UK; State Key Laboratory of Geomicrobiology and Environmental Changes, China University of Geosciences, Wuhan 430074, China; Faculty of Materials Science and Chemistry, China University of Geosciences, Wuhan 430074, China; Department of Earth Sciences, University of Oxford, Oxford OX1 3AN, UK

**Keywords:** Shijiahe civilization, annually laminated stalagmite, calcium isotopes, rainfall reconstruction, culture collapse, 4.2 kyr event

## Abstract

A lack of quantitative rainfall reconstruction has hindered understanding of the role of hydrological disturbances at ∼4.2 kyr BP (1000 years before present) in the collapse of the Shijiahe culture—an advanced Neolithic society in the Middle Yangtze Valley (MYV). We provide a quantitative paleohydrology reconstruction for the period 4.6–3.5 kyr BP by using calcium isotopes, trace elements and δ^13^C from an annually laminated stalagmite from the MYV. Our reconstructed rainfall shows three drier intervals with rainfall of <700 mm/yr (4.36–4.33 kyr BP, 4.23–4.10 kyr BP, 3.57–3.55 kyr BP) and two wetter intervals with rainfall of >1000 mm/yr (3.95–3.84 kyr BP, 3.70–3.59 kyr BP), with suggestions of tripole/dipole rainfall patterns. Combined with archaeological and paleoflood evidence, these data suggest that the Shijiahe culture underwent transformation during drier periods, but abandoned the region when the rainfall was >1000 mm/yr. This robust, multiproxy record demonstrates that water excess could be as problematic as water shortage, even for advanced civilizations, and contributes to understanding hydrological perturbations at ∼4.2 kyr BP.

## Teaser

A significant hydroclimatic shift from prolonged drought to multi-century extreme rainfall around 4,200 years ago likely triggered the collapse of the Shijiahe culturean advanced Neolithic society in the Middle Yangtze Valley. This finding illustrates that water-excess can be as problematic as water-shortage even for advanced civilizations and advances the understanding of regional to global hydrological disturbances during this period.

## INTRODUCTION

Hydroclimates with supportive, stable levels of rainfall are fundamental for societal development, both today and in the past [1]. Shifts to extreme rainfall can lead to societal collapse and migration away from regions of value [2,3]. Improved understanding of the controls on hydrological cycles are especially relevant now, with food production supporting a world population of >8 billion and with human-induced climate warming causing increased storm and rainfall intensities [1,4]. This study uses cave stalagmite measurements to quantify rainfall in the Middle Yangtze Valley (MYV) region of Central China between 4.6 and 3.5 kyr BP (1000 years before present (1950 CE))—a period during which the Shijiahe culture collapsed and migrated out of the MYV [5,6]. The fertile MYV has been, and remains, a key area of food production. In the modern day, >30% of Chinese rice production is supplied by the MYV [7].

The MYV is within the East Asian monsoon climate regime, which is highly seasonal, with ∼70% of the annual rainfall occurring in May–September (∼160 mm/month) and ∼30% of the rainfall occurring in the remaining months (October–April, ∼50 mm/month) [8]. An abnormal surplus or deficit in rainfall occurring around the boreal summer season can cause floods or droughts in the study area. These flood/drought risks were borne out in recent decades, with dozens of large summer flood events and several summer drought events (Table S3). The boreal summer season is the critical period for rice growth in the MYV, with adverse hydrological conditions leading to a severe reduction in rice yields [1].

**Figure 1.**
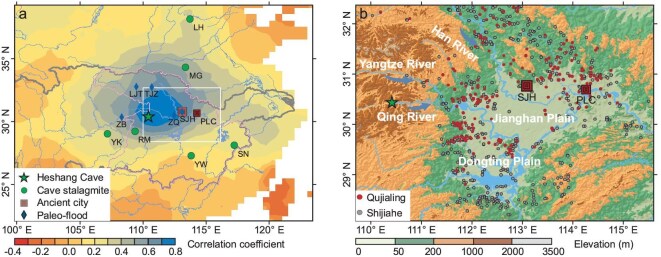
Modern-day climatology of the Yangtze Valley. (a) Star: Heshang Cave (this study, HS). Square: Shijiahe (SJH) and Panlongcheng (PLC) ancient cities. Circle: LH: Lianhua Cave [38]; MG: Magou Cave [39]; RM: Remi Cave [11]; YK: Yangkou Cave [18]; YW: Yuwang Cave [40]; SN: Shennong Cave [2]. Diamond: paleoflood deposits. SJH: Shijiahe [16]; ZB: Zhongba [15]; ZQ: Zhongqiao [14]; LJT: Luojiatan [12]; TJZ: Tuojiazhou [13]. Thin solid line delineates the MYV and thick solid line shows the Yangtze Valley. Rectangle delineates subplot b. Background color denotes modern-day correlation between annual rainfall at HS (from Yichang meteorological station) and the wider region (from CRU TS4.06 rainfall data, for period 1950–2016 [50]), with high correlation between HS and SJH sites (*r* = 0.68, *n* = 67, *P* < 0.001). (b) Circles: Qujialing and Shijiahe archaeological sites. The directions of the arrows indicate the flow of the rivers.

The MYV region has developed multiple successful complex cultures, including the Chengbeixi (∼8.5–7.0 kyr BP), Daxi (∼7.0–5.3 kyr BP), Qujialing (∼5.3–4.6 kyr BP) and Shijiahe (∼4.6–3.9 kyr BP) cultures [9]. Among Neolithic societies, the Shijiahe culture is recognized as the most materially and technologically advanced in this region [5,6]. It has been subdivided into the early-middle stage (∼4.6–4.2 kyr BP) and the late stage (∼4.3–3.9 kyr BP), also called the ‘post-Shijiahe’ [5,6]. Recent archaeology has documented extensive Shijiahe cultural and technological structures, including a core city (∼1.8 km^2^, ‘Shijiahe ancient city’, Fig. 1) with palaces, city walls, advanced water management and sophisticated jade and pottery industries [6] (Fig. S9). However, as documented at multiple sites, the Shijiahe culture began to decline after ∼4.3 kyr BP and collapsed at ∼3.9 kyr BP [5,6]. There is ongoing discussion as to whether the collapse of the Shijiahe culture was caused by raiders from the Central-Plain civilization [5,10] or by changes in climate and rainfall patterns [11]. There is evidence of flooding during the Shijiahe cultural era at multiple excavated sites [12–17] and a growing suggestion of wetter hydroclimate conditions existing at ∼4.2 kyr BP [2,11,18,19]. However, most of these records originated from the broader eastern region of China (105–122°E, 22–43°N), where hydrological conditions exhibit significant spatial heterogeneity (Fig. S8) [20] and few direct and robust paleohydrological records exist in close proximity to the MYV, where the Shijiahe culture thrived [11]. There is also uncertainty regarding the chronology of sedimentary flood horizons [12–17] and the scale and duration of periods of high rainfall.

This article contributes a continuous, quantified record of rainfall from the MYV region, applicable to the Shijiahe culture and more widely to discussions about regional and global hydrological disturbances at ∼4.2 kyr BP.

## RESULTS

### Heshang Speleothem 4.6–3.5 kyr BP hydrological record with precise age model

In this study, we present a high-resolution stalagmite record from Heshang Cave (30°27′N, 110°25′E; 294 m) covering the period 4.6–3.5 kyr BP. Heshang Cave is located immediately west of the MYV, on the banks of the Qing River, which flows into the Yangtze River [21], and on into the Jianghan–Dongting flood plains that constitute the MYV (Fig. 1b). Instrumental data show that the rainfall at Heshang Cave is representative of that of the wider MYV region (Fig. 1a). The speleothem in question, HS4, has been used extensively for hydrological reconstruction studies at key periods in the Holocene [21–23]. HS4 has rapid vertical extension (∼0.3 mm/yr), enabling high-temporal-resolution analysis, and has U concentrations (average ∼495 ppb) that enable accurate and precise absolute chronology [21,22].

Ten U–Th ages specific to the Shijiahe era section of HS4 (Fig. S1 and Table S1) were added to 21 existing U–Th ages spanning the complete Holocene record of HS4 [21]. The average age-model uncertainty is 74 yr (2 s; assessed by using OxCal [24]). Light–dark couplets throughout the Shijiahe era were counted and shown to have occurred at annual resolution, as at other sections of the Heshang record [21–23]. These provide annual layer-counting chronology that was hung on the U–Th age model (Fig. S1). This approach provides relative timing information at close to annual precision (from layer counting) on an absolute age scale (from U–Th). The layer-counting chronology is used to define the duration of unusually wet or dry climate events.

**Figure 2.**
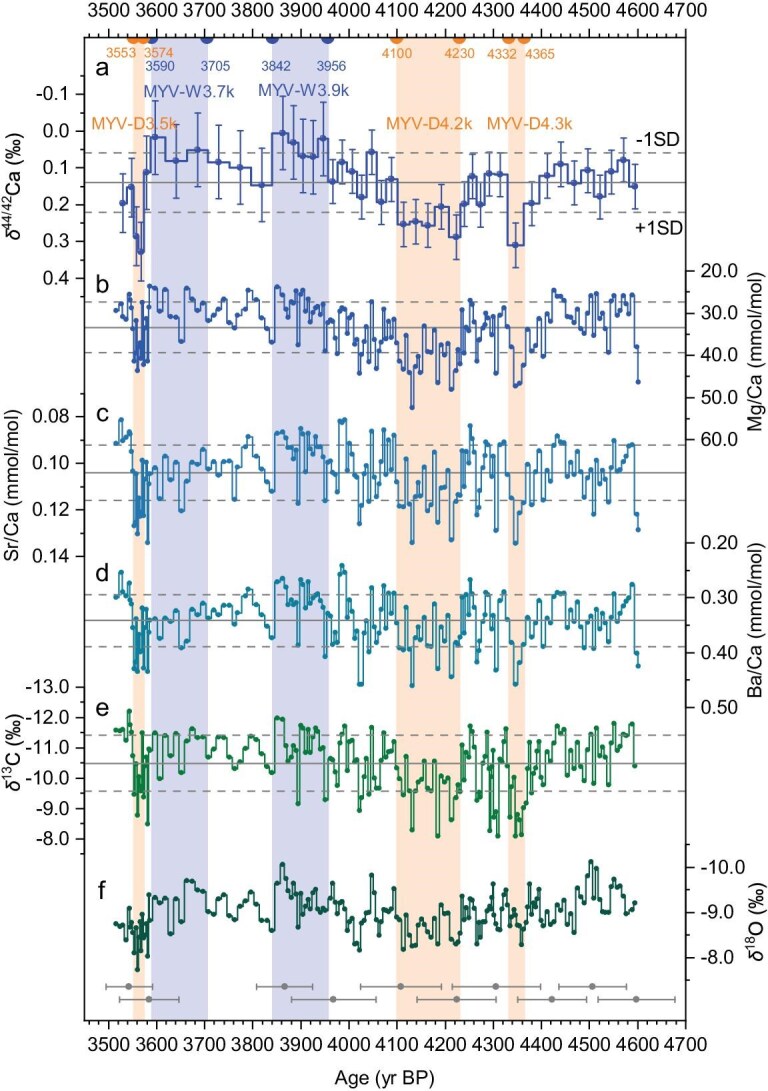
Multiproxy time series from stalagmite HS4 between 4.6 and 3.5 kyr BP. Solid and dashed lines represent average and ±1 SD (standard deviation) values. (a) δ^44/42^Ca with 95% confidence interval error bars. Shaded areas denote drier (wetter) periods defined by δ^44/42^Ca higher (lower) than 1 SD, with three drier periods MYV-D4.3k, MYV-D4.2k and MYV-D3.5k, and two wetter periods MYV-W3.9k and MYV-W3.7k named after the start date of these dry/wet episodes. (b) Mg/Ca_HS4_. (c) Sr/Ca_HS4_. (d) Ba/Ca_HS4_. (e) δ^13^C_HS4_. (f) δ^18^O_HS4_. Ten U–Th age data are shown with ±2 SD uncertainty at the bottom.

Coupled to this absolute chronology, the micro drilling of continuous samples provided 343 CaCO_3_-aliquots, each representing ∼3.5 years of HS4 growth. Every second aliquot was measured for δ^13^C, δ^18^O and X/Ca (where X = Mg, Sr, Ba). Every eighth aliquot was also measured for δ^44/42^Ca, from the same aliquot as for δ^13^C, δ^18^O and X/Ca. All measurements average ∼3.5 years, with measurements every ∼6–7 years for δ^18^O, δ^13^C, Mg/Ca, Sr/Ca and Ba/Ca, and measurements every ∼25 years for δ^44/42^Ca (Fig. 2).

Stable oxygen and carbon isotopes enable direct comparison with a number of existing stable-isotope records. δ^18^O_speleothem_ is controlled by factors including moisture source, evaporation and precipitation amount [25], whilst δ^13^C_speleothem_ is controlled by factors including vegetation (C3/C4 type, vegetation density) and CO_2_ degassing along the flow path and within the cave [2]. These multiple control factors, some of which are non-local, complicate the robust interpretation of stable isotopes. We use δ^44/42^Ca as the cornerstone of our hydrological reconstruction. Speleothem calcium isotopes respond to local, above-cave changes in hydrology [23]. The methodology for using δ^44/42^Ca as a proxy for past absolute rainfall was established at Heshang Cave [23] and verified through the last ∼120 years of instrumental climate data [26]. As rainwater percolates through the karst unsaturated zone, calcite precipitation occurs along the flow path (known as prior calcite precipitation, PCP). Because calcite preferentially incorporates the lighter Ca isotopes during formation, due to the fractionation factor α_solid-solution_ < 1, calcite precipitation causes a systematic increase in δ^44/42^Ca in the fluid phase and subsequent calcite precipitation as PCP proceeds [23] (Sections 4). Based on a simple hydrological model, PCP is proportional to the solution residence time above the cave, which is inversely proportional to the rainfall amount [23] (Fig. S3).

**Figure 3.**
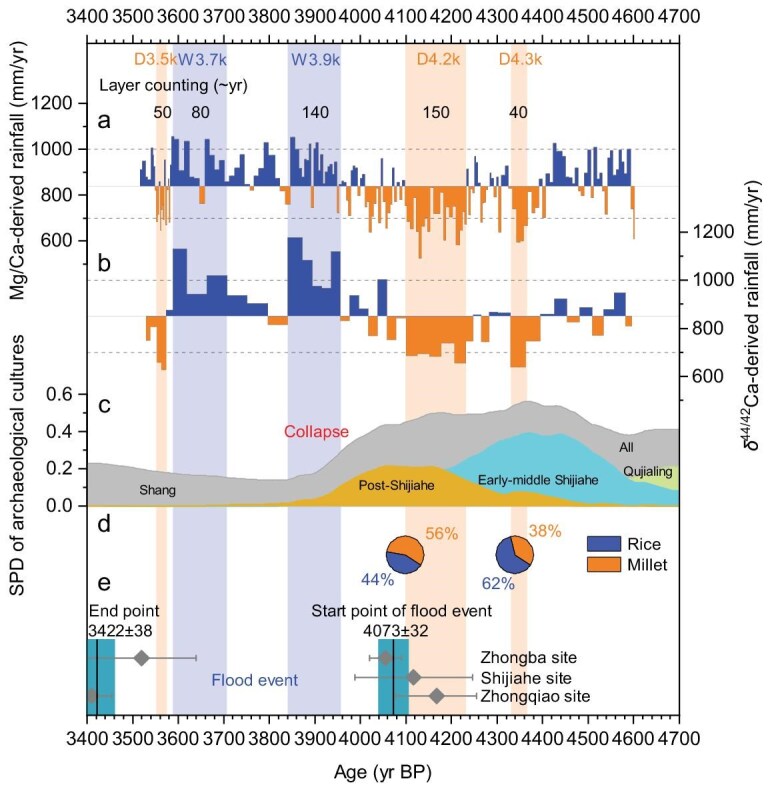
Stalagmite HS4 data alongside MYV archaeology and flood-deposit records. (a) Mg/Ca-derived annual rainfall (this study). (b) δ^44/42^Ca-derived annual rainfall (this study). Dashed lines: reconstructed rainfall of <700 and >1000 mm/yr. Shaded areas: drier and wetter intervals defined by rainfall of <700 and >1000 mm/yr. (c) Summed probability distribution (SPD) analysis of archaeological dates in the MYV. Shaded areas represent all archaeology culture, Qujialing culture, early-middle Shijiahe culture, post-Shijiahe culture from bottom to top, respectively. (d) Pie charts of rice and millet crops from early-middle Shijiahe and post-Shijiahe cultures. (e) Paleoflood events occurring in the MYV for the period ∼4.0 kyr BP. The bars (vertical lines for mean values and widths for ±2 SD uncertainty) showed the start point and end point of the flood event, which were calculated by combining the radiocarbon data (diamonds with ±2 SD uncertainty) of cultural layers above/below the paleoflood layer.

**Figure 4.**
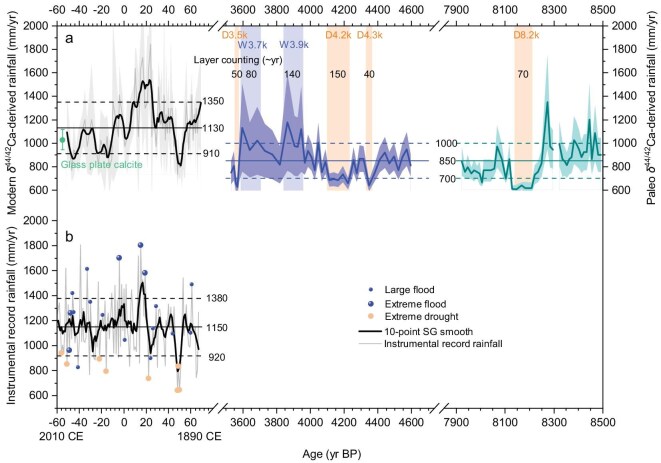
Modern-day, 4.6–3.5 kyr BP and 8.5–7.9 kyr BP rainfall. Solid and dashed horizontal lines: average and ±1 SD rainfall amounts, respectively. (a) Stalagmite HS4 δ^44/42^Ca-derived rainfall for two modern periods, 4.6–3.5 kyr BP and 8.5–7.9 kyr BP. Point on the left side: 2005–2006 CE [23]. Line on the left side: 1883–2001 CE [26]. Line in the middle: 4.6–3.5 kyr BP (this study). Line on the right side: 8.5–7.9 kyr BP [23]. (b) Instrumental rainfall data from Yichang meteorological station (1883–2010 CE). Circles: MYV flood and drought events. Ten-point Savitzky-Golnay (SG) filters (solid lines) are shown.

The fractionation factor used in this study, α^44/42^ = 0.99937± 0.00003, is based on 15 measurements of drip solutions and of corresponding calcite precipitates on glass plates deployed *in situ* at the HS4 stalagmite growth location over a 2-year period [23]. Whilst calcium isotope α_solid-solution_ has been observed to vary between sites [27], we do not expect a significant change in α at the HS4 drip site, for the following reasons. Despite large seasonal variations in cave–air temperatures and drip rates, there was no statistically significant variation in α over the 2-year period during which 15 measurements of α were conducted at the HS4 drip site (*t*-test, 95% confidence interval). The range of solution saturation indices (0.65–1.25) measured at Heshang Cave [28], with corresponding modeled growth rates of 2.3–3.1 × 10^−8^ mmol cm^−2^ s^−1^ [29], is too small to have had a significant impact on the growth rate of α, based on the cave-analogue experiments of Reynard *et al.* [30]. As discussed fully in Owen *et al.* [23], we are assuming that changes in δ^44/42^Ca_speleothem-HS4_ are caused by changes in PCP rather than changes in the isotopic composition of dissolved bedrock. Whilst the isotopic homogeneity (or otherwise) of individual bedrock carbonate formations is poorly constrained, it is unlikely that ∼0.35‰ changes in δ^44/42^Ca_speleothem-HS4_ could be caused by changes in the bedrock composition given the narrow range of seawater values through time and the narrow stratigraphic age range of the rocks overlying Heshang Cave. A source composition control would also not explain the correlations between δ^44/42^Ca and Mg/Ca, Sr/Ca, Ba/Ca and δ^13^C (Fig. S4). Measurements of δ^44/42^Ca every ∼25 years between 4.6 and 3.5 kyr BP provide 43 new values of reconstructed rainfall, each for a period averaging ∼3.5 years. The reconstructed rainfall ranges from 630 to 1180 mm/yr (Fig. 3b). The average 4.6–3.5 kyr BP rainfall amount of 850 mm/yr (1 s, ±140 mm/yr) is lower than the modern-day average rainfall of 1150 mm/yr (1 s, ±230 mm/yr) (Fig. 4).

We also consider trace-element-to-calcium ratios (X/Ca) and δ^13^C as additional hydrological indicators, as per previous Heshang Cave work [8]. When plotted against δ^44/42^Ca, X/Ca and carbon isotopes follow the expected PCP trends (Fig. S4) modeled by using CaveCalc software [31] with partition coefficients from Day and Henderson [32]. This agreement between X/Ca, δ^13^C and expected PCP confirms that trace elements and carbon isotopes are responding (in a relative sense) to changes in hydrology. Measurements of trace elements and δ^13^C at intervals of 6–7 years provide subdecadal hydrological reconstruction (Fig. 2).

This multiproxy HS4 dataset, made up of 925 independent measurements, is interpreted alongside archaeological [9], archaeobotanical [9] and paleoflood datasets (Table S4) [12–16] to fully investigate the timing, duration and amplitude of climate variability over the whole Shijiahe-culture era.

### Timing of high- and low-rainfall periods

We interpret that higher (lower) values of δ^44/42^Ca, X/Ca and δ^13^C correspond to drier (wetter) conditions (Fig. 2) and we pick out key periods for which δ^44/42^Ca is >1 SD above (below) the average δ^44/42^Ca between for the entire 4.6–3.5 kyr BP period, corresponding to an average rainfall of 850 mm/yr. Hydrologically, these anomalous rainfall periods correspond to times at which the rainfall was <700 mm/yr or >1000 mm/yr. Based on this criterion, there are three drier periods (4.36–4.33, 4.23–4.10 and 3.57–3.55 kyr BP) and two wetter periods (3.95–3.84 and 3.70–3.59 kyr BP) (Fig. 2). For ease of reference, the three drier climate periods are called ‘D4.3k’, ‘D4.2k’ and ‘D3.5k’, and the two wetter periods are called ‘W3.9k’ and ‘W3.7k’, based on the start date of the hydroclimate transition in 1000 years before 1950 CE. Where necessary, the prefix ‘MYV-’ is added to clarify the location. The multi-decade durations of these events based on the layer-counting chronology (from 40 to 150 years’ duration) are shown in Fig. 3.

## DISCUSSION

### Rainfall trends and archaeology in the MYV at ∼4.2 kyr BP

**Figure 5.**
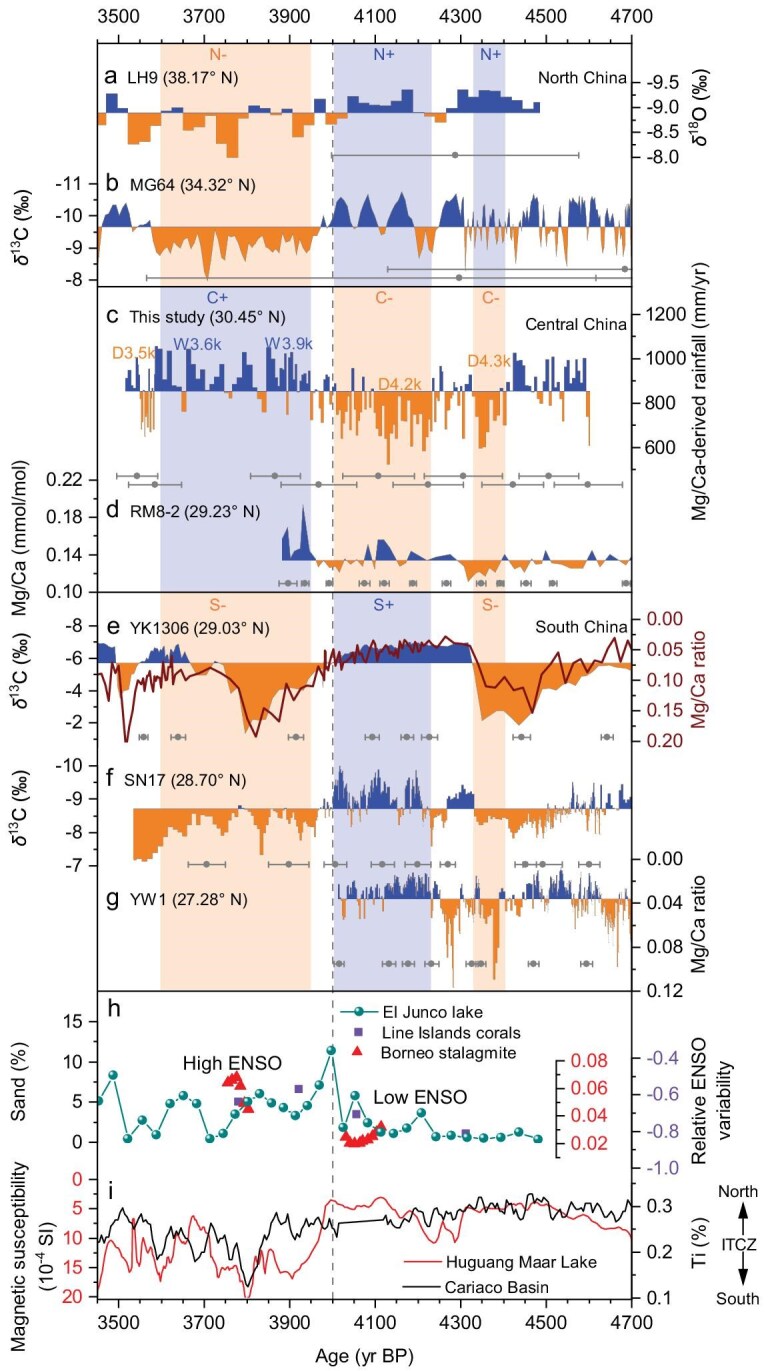
Reconstruction of hydroclimate in the MYV compared with other hydrological changes in East China (a–g), with (h) relative ENSO variability and (i) migration of the Intertropical Convergence Zone (ITCZ). (a) Stalagmite δ^18^O record from Lianhua Cave [38]. (b) Stalagmite δ^13^C record from Magou Cave [39]. (c) Stalagmite HS4 Mg/Ca-derived rainfall for 4.6–3.5 kyr BP (this study). (d) Stalagmite Mg/Ca from Remi Cave [11]. (e) Stalagmite δ^13^C and Mg/Ca ratio from Yangkou Cave [18]. (f) Stalagmite δ^13^C from Shennong Cave [2]. (g) Stalagmite Mg/Ca ratio from Yuwang Cave [40]. (h) ENSO variability calculated from stalagmite BA03 δ^18^O records (triangle) in Borneo [45] and Line Islands fossil coral δ^18^O records (square) in the Central Pacific compiled by Chen *et al.* [44] and from El Junco Lake Sand percentage (circle) in the Eastern tropic Pacific [41]. (i) Magnetic susceptibility in the Huguang Maar Lake in Southern China [46] and Ti content from the Cariaco Basin [47]. Different color configurations of the vertical bars reflect the spatial heterogeneities in precipitation in East China. Bars and alphabets with negative signs (N–/C–/S–) represent low rainfall, while the bars and alphabets with positive signs (N+/C+/S+) suggest high rainfall. N, C and S are the abbreviations for ‘North China’, ‘Central China’ and ‘South China’, respectively. The detailed positions of the above records can be found in Fig. 1a. U–Th and ^14^C age data are shown below paleohydrology records with ±2 SD uncertainty.

Between 4.6 and 3.5 kyr BP, we see evidence of two wetter periods (W3.9k, W3.7k), which lasted for ∼140 and ∼80 years, respectively. These reconstructed wet periods are consistent with the pluvial conditions from other regional paleohydroclimatic records [11] (Fig. 5d) and findings of sedimentary studies, which suggest that MYV flooding occurred sometime during the period 4.07–3.42 kyr BP [14–16] (Figs 1a and 3e). Two additional studies, using optically stimulated luminescence dating, reported river overbank flooding earlier, between 4.2 and 4.0 kyr BP [12,13] (Fig. 1a). A speleothem record from Remi Cave, ∼1° south of Heshang Cave, reported pluvial pulses at 4.25–4.12 and 4.0–3.9 kyr BP (Fig. 5d), with the suggestion of an adverse effect on low-lying settlements [11]. Significant lake expansion in the Jianghan–Dongting plain [17] and increases in hygrophyte pollen at the Shijiahe ancient city are indicative of marshy, wetland conditions [16]. Increased surface water in the MYV plains will have reduced the available space for habitations and food production. The high-resolution nature and precise chronology of our speleothem record now enables an accurate assessment of the duration of these wet periods, based on the combined use of hydrological proxies and annual lamina counting. The wettest periods (>1000 mm/yr of rainfall), which we presume caused the extensively reported floods, lasted for up to 50 years during the 140- and 80-year-long wet periods W3.9k and W3.7k (Fig. 3). The multidecadal duration of these events is likely to have driven people to higher, less flood-prone areas. Expansive agricultural land is particularly difficult to protect from flooding.

There is a clear relationship between the periods of high rainfall observed in our new record and the periods of decline in the Shijiahe culture. We use the summed probability distribution of archaeological artifacts to assess Shijiahe population patterns through time [9] (Fig. 3 and Fig. S7). The population in the MYV, which peaked at 4.4–4.1 kyr BP, dropped rapidly from the start of W3.9k. The population remained low for centuries throughout W3.9k and W3.7k. The overall picture is of high rainfall (>1000 mm/yr) causing flooding, extensive wetlands and a decreased MYV population. We use our new information on rainfall amounts and wet-period durations to suggest that hydrology had a direct impact on the decline and ultimate collapse of the Shijiahe (Fig. 3). This fits with suggestions of post-Shijiahe people abandoning their city and migrating to hills above the Jianghan–Dongting floodplain or farther afield [6,11].

The new record indicates that the impact of dry periods on the Shijiahe culture was rather less pronounced. The first two drier events, D4.3k and D4.2k, agree with findings from previous sediment and pollen studies with lower age precision, which reported contracting lake areas, a lowering of the groundwater table [17], a significant rise in the number of xerophilous herbs and a decline in broad-leaved trees during this period [10]. There is also agreement with the Remi Cave speleothem record that suggested drier conditions until ∼4.0 kyr BP [11] (Fig. 5d). Archaeobotanical data of macroremains and of phytoliths have shown that the proportion of cultivated rice reduced significantly relative to that of cultivated millet during this period (Fig. 3d). Millet is a short-season, xerophytic crop with optimal rainfall intervals ranging from 350 to 550 mm [9]. The shift to increased millet production suggests successful adaptation to a drier environment, with high population levels maintained throughout the D4.3k and D4.2k periods (Fig. 3). There is, however, evidence of a cultural transition occurring at this time (∼4.3–4.0 kyr BP), with the post-Shijiahe culture increasingly taking over from the early-middle Shijiahe culture (Fig. 3). There is archaeological evidence to support this transition, with the preservation of some early-middle Shijiahe culture alongside the incorporation of substantial external cultural influences from the Central-Plain area [6,11]. The post-Shijiahe culture featured advanced jade production influenced by Central-Plain cultures, within less complex, more sparsely populated societies [6,11].

The third drier period observed in our record (D3.5k) is coeval with a new civilization: the Shang Dynasty, which established itself in the MYV with a notable site at the ancient city of Panlongcheng [33]. Archaeological evidence confirms that the water levels in rivers and lakes surrounding the Panlongcheng ruins were substantially lower than present-day levels [33]. Overall, we see three separate periods (D4.3k, D4.2k and D3.5k) within which lower rainfall (∼750 mm/yr) is coeval with cultural developments in the MYV and migrations into the area, in contrast to periods of high rainfall, which we argue contributed to the collapse of the post-Shijiahe culture.

The Ca-isotope-derived hydroclimate information discussed above is also evident in the Mg/Ca data, which provide a less quantitative but higher-resolution record of rainfall, with a Mg/Ca measurement every ∼6–7 years of HS4 growth. The Mg/Ca response to rainfall was calibrated with the δ^44/42^Ca-derived rainfall (Fig. S5, Sections 5 and Fig. 3a). The higher-resolution Mg/Ca dataset supports the durations of high and low rainfall assessed from Ca isotopes and does not change our interpretations of the impacts of rainfall on MYV cultures. The wettest events (based on δ^44/42^Ca or Mg/Ca) occur during the W3.9k and W3.7k periods, after the post-Shijiahe culture had moved away from the MYV (Fig. 3). The driest events, including an ∼35-year spell of rainfall of <700 mm/yr during D4.2k, appear not to have affected the population size, perhaps because of prior adaptation to the increased growth of millet compatible with the rainfall of 350–550 mm/yr [9] (Fig. 3).

### Comparison of MYV rainfall at ∼4.2 kyr BP with other periods and locations

Our reconstructed average and highest rainfall amounts from 4.6 to 3.5 kyr BP (∼850 and 1200 mm/yr) are lower than the average and maximum yearly rainfall amounts recorded over the past 120 years (∼1150 and 1500 mm/yr) (Fig. 4). Without modern water-management technology, Neolithic cultures were forced to migrate out of the fertile MYV region. In contrast, it is a measure of the success of modern society that current water-management practices and double-cropping rice production enable the MYV to account for >30% of the Chinese output of rice [7,34]. In the future, with rising temperatures, the intensity of droughts and floods is expected to increase [4], requiring continued assessment of the region’s hydrology to ensure effective water- and agricultural-management decisions.

Rainfall reconstructions from HS4 are also available for the period around the 8.2-kyr event [22,23] (Fig. 4). δ^44/42^Ca-derived rainfall is ∼1000 mm/yr at 8.5–8.3 kyr BP, with an ∼70-year drop to ∼600 mm/yr at 8.2 kyr BP, before increasing to ∼800 mm/yr. Based on the impact of wet periods (W3.9k and W3.7k) at ∼4.2 kyr, we might expect that the high rainfall early in this record may have led to flooding and challenging conditions faced by societies present in the MYV at that time.

Consideration of our new record alongside existing paleohydrology records from China and beyond provides important input for researching the complex processes underlying observed hydrological change. It is, at first, surprising that, for most of the period of 4.6–3.5 kyr BP, our record has opposing hydrological trends to those of the Yangkou and Shennong Caves located only 390 and 690 km away from Heshang Cave [2,18] (Figs 1a and 5). But analysis of modern-day climate records [20] shows the same trends in recent decades (Fig. S8), i.e. when Heshang Cave is subjected to anomalously dry conditions, Yangkou and Shennong Caves are anomalously wet and vice versa. A growing number of modern-day meteorological studies [20,35] and palaeo-reconstruction from the last millennium [25] identify tripole and dipole rainfall patterns throughout Eastern China that help to explain the East Asian summer monsoon (EASM) rainfall trends. Tripole patterns of the form N+/C–/S+ (and N–/C+/S–) denote anomalously high rainfall in Northern (N) and Southern China (S), along with anomalously low rainfall in Central China (C) (and vice versa). Dipole patterns (‘N+/S–’ and ‘N–/S+’) have also been proposed, with opposing anomalous rainfall north and south of the Yangtze River valley [20,25,35]. Some of the many control factors underlying these rainfall patterns have been identified as: the strength and position of the Western Pacific Subtropical High (WPSH) pressure system (Fig. S8); the state of climate oscillations such as the El Niño Southern Oscillation (ENSO), the Pacific Decadal Oscillation (PDO) and the Atlantic Multidecadal Oscillations (AMO); the position of the Meiyu-front and of the East Asian monsoon trough; and the strength and position of the East Asian Jet [20,25,35,36]. Of these factors, the ENSO and the WPSH are suggested to be of high significance [20,36]. Without hypothesizing on the complex underlying climate dynamics [3,37], we summarize some key observations from our results. Heshang Cave speleothem records provide strategically located hydrological data within the Central China (C) region of the tripole and dipole rainfall patterns that are increasingly researched. Time-series analysis of our ∼6–7 year resolution trace-element results reveals statistically significant (99% confidence interval) periodicities at ∼27 and ∼66 years, typical of PDO and AMO oscillations, respectively (Fig. S6). The combined data from a meridional transect of hydrology-focused speleothem records [2,11,18,38–40] suggest a ‘N+/C–/S+’ tripole pattern of the EASM between ∼4.3 and 4.0 kyr BP, switching to the opposite ‘N–/C+/S–’ pattern between ∼3.9 and 3.6 kyr BP, and a likely ‘N+/S–’ dipole between ∼4.4 and 4.3 kyr BP (Fig. 5 and Fig. S8). The timing of the switch from ‘N+/C–/S+’ to ‘N–/C+/S–’, at ∼4.0 kyr BP, is also likely to be significant (Fig. 5). It occurs at the same time as an increasingly well-documented shift from low to high ENSO variability [41–45] and of a likely southwards shift in the position of the Intertropical Convergence Zone [46,47] (Fig. 5). This ENSO shift signifies the end of a long period of low ENSO variability marked by a ‘La Niña-like’ state in the mid-Holocene, supported by models and paleo-records alike [41–45]. Overall, our new quantitative rainfall record, with precise chronology, identifies the hydrological conditions associated with cultural developments within the MYV and can help to connect local hydrology with larger-scale factors such as the WPSH, ENSO, PDO and AMO.

## MATERIALS AND METHODS

All U–Th, δ^44/42^Ca, δ^18^O, δ^13^C, and trace-element measurements were carried out at the University of Oxford, Department of Earth Sciences.

### U–Th ages

Calcite blocks (∼0.35 g) were sampled with a 0.9-mm dentil bur along the growth axis of stalagmite HS4 (Fig. S1). Samples were dissolved, spiked with a mixed ^229^Th–^2^^36^ U spike, purified by using ion-exchange chemistry and analysed by using a Nu Instruments multicollector inductively coupled plasma mass spectrometer (MC-ICP-MS) [48]. Raw ages were corrected for initial ^230^Th content by using the measured ^232^Th content and assuming an initial (^230^Th/^232^Th) = 1.97 (based on a measurement of the present-day drip-water chemistry [21]). Uncertainty in this correction is assumed to be 50% of the size of the correction.

### Age model and annual lamination

The age model with 68%, 95% confidence ranges was produced by using the OxCal Version 4.4 Poisson process deposition model [*k*_0_ = 1 cm^−1^, log_10_(*k*/*k*_0_) = U(−2,2)], with interpolation [24] (Fig. S1 and Table S2). Visible light–dark couplet growth laminae were verified as the annual lamination based on U–Th chronology (Fig. S1 and Table S2), with laminae counting used to establish the duration of wet and dry periods (Fig. 3).

### Proxy sample milling

The working half of HS4 was slabbed. Micro drilling of 343 continuous samples was undertaken by using a New-Wave Micromill. The 900-μm-diameter tungsten carbide dental bit was cleaned with ethanol between samples. Sample trenches followed growth banding at 900-μm intervals. Each trench was 2 mm long along the growth band and 300 μm deep. The top 50 μm of each trench was discarded to remove surface contamination. Every second sample was measured for δ^13^C, δ^18^O and X/Ca. Every eighth sample was also measured for δ^44/42^Ca, from the same aliquot as for δ^13^C, δ^18^O and X/Ca. Each measurement averages ∼3.5 years of HS4 growth, with measurements every 6–7 years for δ^13^C, δ^18^O and X/Ca, and ∼25 years for δ^44/42^Ca (Dataset S3).

### Calcium-isotope ratios

Aliquots of ∼500 μg of CaCO_3_ were dissolved in 2 M distilled HNO_3_. Automated Ca–Sr separation (PrepFAST MC, Elemental Scientific, USA) was used to separate the Ca from the Sr, Mg and other matrix elements to avoid isobaric interferences during analyses on a Nu Instruments MC–ICP–MS [23]. δ^44/42^Ca is reported normalized to NIST SRM 915a, a standard reference material (SRM) from the National Institute of Standards and Technology (NIST). The measured value for our purified SRM 915B was δ^44/40^Ca = 0.76 ± 0.08‰ (2 s, *n* = 27), which matches the values obtained by using a thermal ionization mass spectrometer (TIMS), at δ^44/40^Ca = 0.72 ± 0.04‰ [49].

### Stable-isotope ratios

Aliquots of ∼60 μg of CaCO_3_ were measured by using a Delta V Advantage mass spectrometer coupled to a Kiel IV carbonate device. Samples were reacted with 100% H_3_PO_4_ at 71°C, calibrated with NBS-18, NBS-19 bracketing standards. The relative ^13^C/^12^C and ^18^O/^16^O values are reported by using conventional ‰ notation on a scale with δ^18^O of NBS-19 = –2.2‰. Standard deviations are δ^18^O = 0.09‰ and δ^13^C = 0.04‰ (1 s). The higher-resolution records overlap with previous lower-resolution measurements (Fig. S2).

### Trace-element measurements

Samples were analysed by using a PerkinElmer NexIon 350D quadrupole ICP–MS with a universal cell. We report Mg/Ca, Sr/Ca and Ba/Ca with external precision (2 s) of 3.1%, 3.8% and 4.4%, respectively, quantified by using a secondary standard interspersed throughout the analyses.

## Supplementary Material

nwaf567_Supplemental_Files
