# Supplementary material for: Precise chronology of hydrological changes at ∼4.2 kyr in Central China to assess the impact of flooding on Neolithic societies
Source: Natl Sci Rev. 2025 Dec 11;13(2):nwaf567. doi: 10.1093/nsr/nwaf567 (PMC12839523; doi:10.1093/nsr/nwaf567)
Supplement: nwaf567_Supplemental_Files [file nwaf567_supplemental_files.zip › SuppInfo_ok.docx]

**Supporting Information**

**Precise chronology of hydrological changes around 4.2kyr in central China to assess the impact of flooding on neolithic societies**

Jin Liao^1, 2^, Christopher C. Day^3*^, Chaoyong Hu^1^, Yuhui Liu^4^, and Gideon M. Henderson^3^

^1^State Key Laboratory of Geomicrobiology and Environmental Changes, China University of Geosciences, Wuhan, 430074, China

^2^Wuhan Center, China Geological Survey, Wuhan, 430205, China

^3^Department of Earth Sciences, University of Oxford, Oxford, OX1 3AN, UK

^4^Faculty of Materials Science and Chemistry, China University of Geosciences, Wuhan, 430074, China

***Correspondence author**. Email: chris.day@earth.ox.ac.uk (C.C. Day)

**Keywords:** Shijiahe civilization; Annually laminated stalagmite; Calcium isotopes; Rainfall reconstruction; culture collapse; 4.2 kyr event

**I) Heshang cave, stalagmite HS4 data (this study)**

**1) Stalagmite HS4 U-Th chronology**

**Table S1.** U and Th data for stalagmite HS4 chronology. Distance is the midpoint distance from the top of the stalagmite, with smaller distances corresponding to younger ages. Measured isotope ratios are given in activity format, e.g. (234/238) = (N_234_*λ_234_)/(N_238_*λ_238_). Ages were calculated using half-lives from [[1](#_ENREF_1)]. Corrected ages are corrected for initial ^230^Th using the measured ^232^Th content and assuming an initial (^232^Th/^230^Th) = 1.97 (a value based on measurement of the present-day drip water chemistry [[2](#_ENREF_2)]). Uncertainty in this correction is assumed to be 50% of the size of the correction.

| **Stal ID** | **Distance**  **(cm)** | **238U conc**  **(ppm)** | **(230/232)** | **(234/238)** | **(234/238)**  **error**  **(2s)** | **(230/238)** | **(230/238)**  **error**  **(2s)** | **(232/238)** | **(232/238)**  **error**  **(2s)** | **Raw Age**  **(yr BP)** | **Corr. Age**  **(yr BP)** | **Corr. Age**  **Error**  **(2s)** |
| --- | --- | --- | --- | --- | --- | --- | --- | --- | --- | --- | --- | --- |
| HS4-1 | 147.20 | 0.046 | 50.85 | 1.79 | 7.33E-03 | 0.078 | 1.31E-03 | 1.54E-03 | 1.05E-05 | 4798 | 4614 | 92 |
| HS4-2 | 144.40 | 0.040 | 50.95 | 1.81 | 7.26E-03 | 0.078 | 9.16E-04 | 1.52E-03 | 8.50E-06 | 4694 | 4514 | 90 |
| HS4-3 | 142.18 | 0.056 | 50.02 | 1.81 | 7.25E-03 | 0.076 | 1.01E-03 | 1.52E-03 | 1.01E-05 | 4602 | 4422 | 90 |
| HS4-4 | 139.44 | 0.068 | 29.08 | 1.82 | 7.06E-03 | 0.075 | 8.45E-04 | 2.59E-03 | 1.43E-05 | 4521 | 4217 | 152 |
| HS4-5 | 136.05 | 0.133 | 35.52 | 1.80 | 8.36E-03 | 0.074 | 7.57E-04 | 2.09E-03 | 9.46E-06 | 4508 | 4260 | 124 |
| HS4-6 | 132.56 | 0.082 | 35.33 | 1.80 | 7.85E-03 | 0.072 | 7.85E-04 | 2.02E-03 | 1.14E-05 | 4330 | 4090 | 120 |
| HS4-7 | 127.61 | 0.136 | 25.74 | 1.81 | 6.91E-03 | 0.071 | 8.15E-04 | 2.76E-03 | 1.25E-05 | 4295 | 3968 | 163 |
| HS4-8 | 123.86 | 0.054 | 67.52 | 1.82 | 7.14E-03 | 0.067 | 8.39E-04 | 9.90E-04 | 7.37E-06 | 3999 | 3883 | 58 |
| HS4-9 | 119.27 | 0.098 | 51.17 | 1.82 | 8.42E-03 | 0.061 | 6.11E-04 | 1.20E-03 | 6.22E-06 | 3658 | 3517 | 70 |
| HS4-10 | 116.02 | 0.049 | 135.20 | 1.81 | 7.18E-03 | 0.060 | 9.77E-04 | 4.47E-04 | 6.08E-06 | 3628 | 3575 | 60 |

**2) Age model and annual-growth laminae**

Stratigraphic locations (Distance (cm)) and initial-Th corrected U-Th ages were input into OxCal V4.4 to produce a calcite-deposition age model (Table S2). The OxCal Poisson process deposition model was used for this age model with input parameters (k_0_ = 1 cm^−1^, log10(k/k_0_) = U(−2,2), [[3](#_ENREF_3)]) (Fig. S1 and Table S2). Visible light-dark couplet growth laminae were verified as being annual, based on comparison with U-Th chronology (Fig. S1). Layer-counted ages were fitted to the OxCal age model in such a way as to minimise deviation between average OxCal and average layer-counted ages, with results in Table S2 and Fig. S1. The chronology of all measurements (isotopes, trace-elements) is based on the OxCal fitted layer-counted ages.

**Table S2.** Oxcal version 4.4 modelled ages using Poisson-process deposition model (k_0_ = 1cm^-1^, log_10_(k/k_0_) = U(-2,2)).

| **Stal ID** | **Distance (cm)** | **Oxcal modelled ages (yr BP)** | | | | | | **Layer counted age (yr BP)** |
| --- | --- | --- | --- | --- | --- | --- | --- | --- |
|  |  | **mu** | **sigma** | **68% CI** | | **95% CI** | |  |
| HS4-1 | 147.20 | 4598 | 40 | 4637 | 4557 | 4680 | 4518 | 4622 |
| HS4-2 | 144.40 | 4506 | 35 | 4541 | 4469 | 4576 | 4437 | 4536 |
| HS4-3 | 142.18 | 4422 | 36 | 4459 | 4388 | 4494 | 4350 | 4457 |
| HS4-4 | 139.44 | 4306 | 46 | 4358 | 4264 | 4396 | 4210 | 4344 |
| HS4-5 | 136.05 | 4224 | 41 | 4266 | 4183 | 4309 | 4139 | 4222 |
| HS4-6 | 132.56 | 4108 | 42 | 4152 | 4067 | 4194 | 4022 | 4088 |
| HS4-7 | 127.61 | 3968 | 44 | 4009 | 3919 | 4058 | 3882 | 3944 |
| HS4-8 | 123.86 | 3866 | 29 | 3896 | 3835 | 3925 | 3806 | 3800 |
| HS4-9 | 119.27 | 3585 | 31 | 3612 | 3551 | 3649 | 3526 | 3614 |
| HS4-10 | 116.02 | 3543 | 24 | 3568 | 3518 | 3592 | 3493 | 3507 |

**Figure S1.** Age models and slabbed sections of stalagmite HS4. (a) Light blue: U/Th ages corrected for initial ^230^Th with vertical bars representing 2s uncertainty. Dark blue: OxCal ages with 2s uncertainty using OxCal software version 4.4 Poisson process deposition model [k_0_ = 1 cm^−1^, log_10_(k/k_0_) = U(−2,2)], with interpolation [[3](#_ENREF_3)]. Red line: layer-counted ages fitted to the OxCal age model. (b) HS4 age versus depth from OxCal Poisson process deposition age model with 68% (dark blue) and 95% (pale blue) confidence ranges. White circles: original U-Th sample ages with error bars. Individual sample age distributions are shown in dark grey (68%) and light gray (95%). (c) Sedimentary characteristics and laminations of stalagmite HS4 (4.6-3.5 kyr BP). Numbers along the left and right edges denote age (in years BP) and depth respectively.


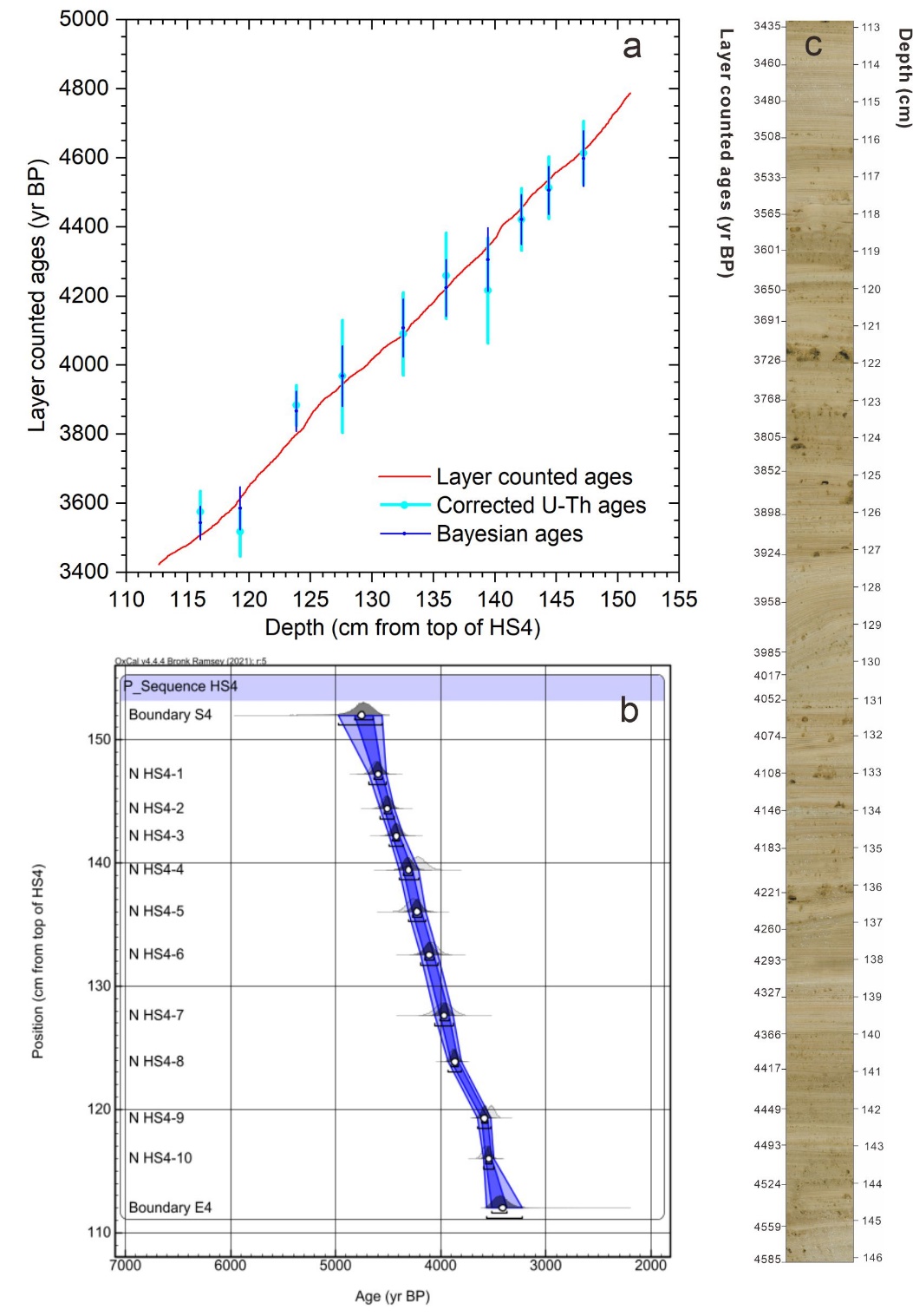


**3) Stable isotope data**

**Figure S2.** Stalagmite HS4 δ^18^O and δ^13^C at two sampling resolutions. Black curves: ~20 years/sample resolution from [[2](#_ENREF_2)]. Red curves: 6~7 years/measurement resolution (this study).


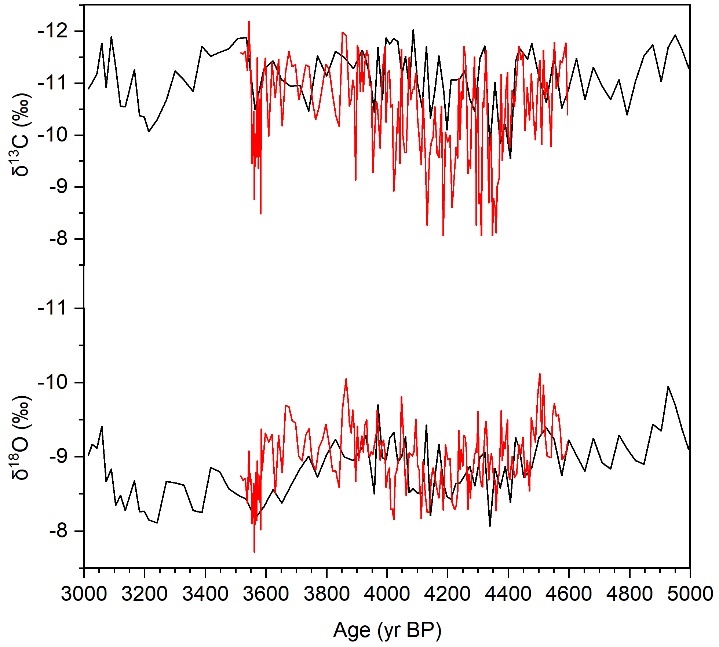


**4) Summary of equations, constants and fractionation factors used for δ^44/42^Ca-derived rainfall reconstruction**

This study uses the same methodology for δ^44/42^Ca-derived rainfall reconstruction as published in Owen et al. [[4](#_ENREF_4)]. We provide a brief summary of the underlying equations, constants and fractionation factors employed. For a more detailed explanation c.f. Owen et al.[[4](#_ENREF_4)]. There are three main parts to the process of reconstructing rainfall with Ca-isotopes: i) Calibration of the cave’s Ca-isotopes and of modern-day rainfall, for feeding into a hydrological model; ii) Ca-isotopes from stalagmite growth laminae are used to reconstruct absolute Prior Calcite Precipitation (PCP) for periods in the past; iii) PCP is fed into a simple hydrological model to derive annual rainfall amount (mm/year). In subsequent text *f* is the fraction of dissolved Ca remaining in solution, with e.g. *f* = 0.9 indicative of 90% of dissolved Ca remaining in solution.

Calibration of the Heshang cave system for Ca-isotopes was conducted over two years, with fifteen coupled solution, CaCO_3_ measurements (hereafter $\text{r}_{\text{d}}$ and $\text{r}_{\text{s}}$ respectively), with CaCO_3_ grown on glass plates under the HS4 stalagmite drip [[4](#_ENREF_4)]. The fractionation factor $\text{α}_{\text{solid-solution}}\text{= }\frac{\text{r}_{\text{s}}}{\text{r}_{\text{d}}}\text{ = 0.99937 ± 0.00003}$. The Ca isotopic composition of the initial solution ($\text{r}_{\text{0}}$) (at *ꬵ* = 1) was derived from bedrock measurements, with $\text{r}_{\text{0}}$ = 1.0004 ± 0.00007. As PCP proceeds, the isotopic composition of the solution evolves according to the Rayleigh fractionation equation $\text{r}_{\text{d}}\text{ =}\text{r}_{\text{0}}\text{* }\text{ꬵ}^{\text{ α-1}}$. Substitution and rearrangement gives $\text{ꬵ =}\text{ }{\frac{\text{r}_{\text{s}}}{\text{r}_{\text{0}}\text{*}\text{α}}}^{\frac{\text{1}}{\text{α-1}}}$. Each stalagmite δ^44/42^Ca measurement enables the calculation of $\text{r}_{\text{s}}$, of *f*, and therefore of PCP (PCP = 1 - *f*), for the corresponding growth horizon: $\text{δ}^{\text{44/42}}\text{Ca = }\frac{\text{r}_{\text{s}}}{\text{1000}}$ -1.

A one-box model of the aquifer above the cave gives the residence time of solution above the cave as the ratio of the aquifer volume to the flux of water added to the aquifer by rainwater infiltration (Fig. S3). Here, the infiltration flux is considered proportional to local rainfall. We assume that the extent of PCP is proportional to this residence time. Under these assumptions the extent of PCP, calculated from Ca isotopes, is inversely proportional to effective rainfall (Fig. S3).

PCP = *kR_t_*, $\text{PCP =}\frac{\text{kV}}{\text{rainfall}}$ , with *k* (proportionality constant) and *V* (aquifer volume) both constants, *μ* = *kV*, $\text{r}\text{ainfall = }\frac{\text{μ}}{\text{PCP}}$. Calibrated with the last ~120 years of instrumental climate data and PCP (derived from Ca-isotope measurements of 82 CaCO_3_ subsamples from the top of the Heshang laminated stalagmite over the past 120 years (1881~2001 CE)) [[5](#_ENREF_5)], *μ* = 1148mm/year × 0.32 = 368mm/year.


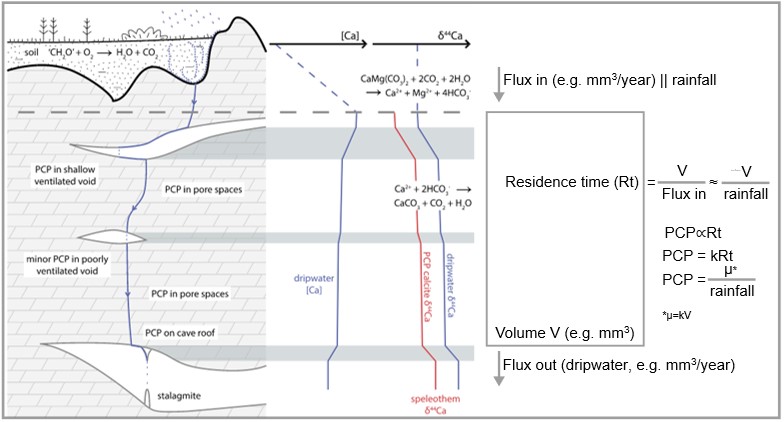


**Figure S3:** Cartoon representation of the one-box model of the aquifer above the cave that is used for δ^44/42^Ca-derived rainfall reconstruction [[4](#_ENREF_4)].


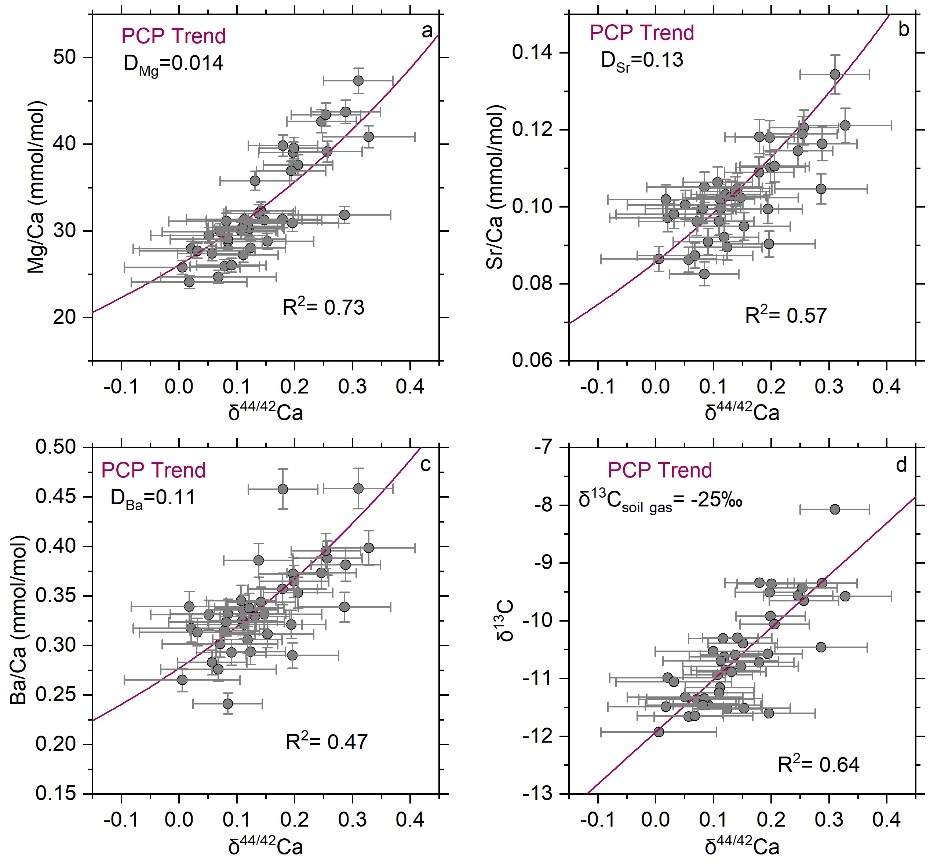


**Figure S44.** Mg/Ca, Sr/Ca, Ba/Ca and δ^13^C plotted against δ^44/42^Ca for the period 4.6-3.5 kyr BP. Red curves: model PCP trends, using methods from Owen et al. [[4](#_ENREF_4),[6](#_ENREF_6)], and partition coefficients from Day and Henderson [[7](#_ENREF_7)] calculated at 19 °C. Initial solution values of Mg/Ca, Sr/Ca and Ba/Ca ratios are fitted to the dataset, with corresponding initial solution X/Ca ratios Mg/Ca_0_ = 1.30 mol/mol, Sr/ Ca_0_ = 0.48 mmol/mol, Ba/Ca_0_ = 1.82 mmol/mol.

**5) Mg/Ca hydrological information**

The principle method for rainfall reconstruction that we use is the established calcium isotope methodology. Ca-isotope derived hydroclimate information is also evident in Mg/Ca, which provides a higher resolution record of rainfall, and supports the durations of high and low rainfall assessed from Ca isotopes. The correlation between Mg/Ca_HS4_ and δ^44/42^Ca_HS4_ is R^2^ = 0.73 (Fig. S5). To ease the comparison of both datasets we establish a logarithmic regression between Mg/Ca and the δ^44/42^Ca-inferred rainfall record, to display Mg/Ca both in terms of units of mmol/mol (Fig. 2), and rainfall mm/year calibrated with δ^44/42^Ca-inferred rainfall (Fig. 3). In all cases there is very close agreement between δ^44/42^Ca and Mg/Ca.


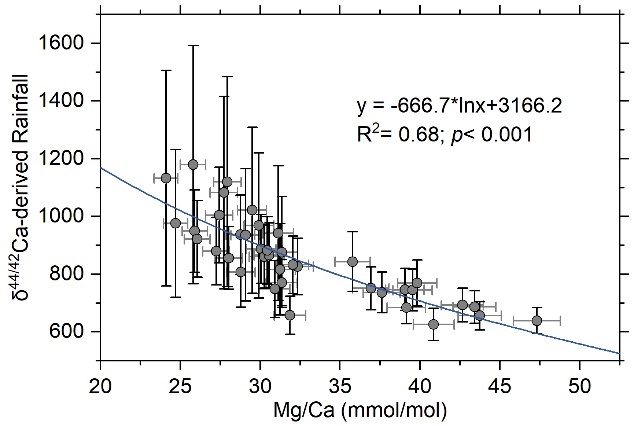


**Figure S5.** Mg/Ca plotted against δ^44/42^Ca-derived rainfall for the period 4.6-3.5 kyr BP.

**6) Timeseries analysis of Mg/Ca_HS4_ records**


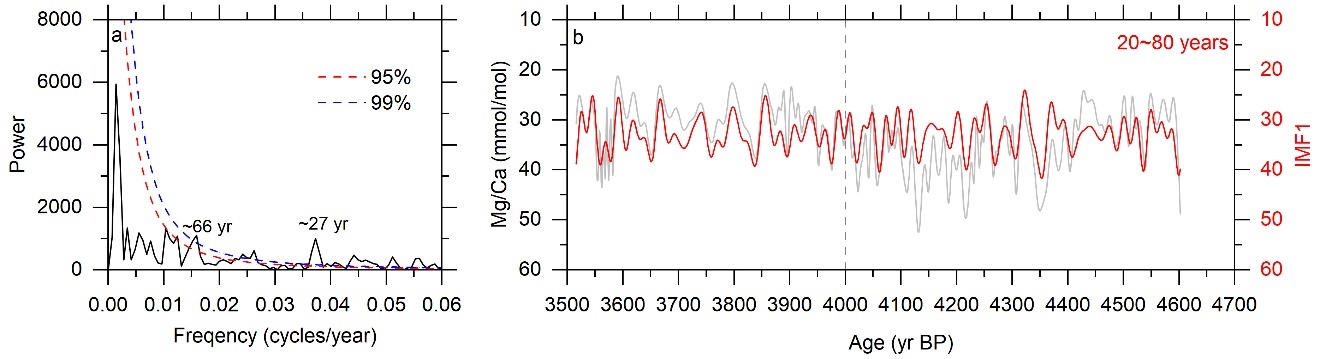
**Figure S6.** Power spectra (a) and bandpass filtering (b) of Mg/Ca_HS4_ records for the period 4.6-3.5 kyr BP. The red and blue dashed lines show the 95% and 99% confidence limits of the power spectra, respectively. Power spectra and bandpass analysis are conducted using the Past software (V4.11) and OriginPro 2022, respectively.

**II) Literature data used in this study**

**1) Major floods and droughts (1889-2022 CE)**

**Table S3.** Annual rainfall amount associated with large flood and drought events recorded by Yichang meteorological stations for the period 1880-2022 CE.

| **Year** | | **Flood/Drought Description** | **Annual rainfall (mm)** | **References** | **Mean rainfall amount (±1s, mm)** |
| --- | --- | --- | --- | --- | --- |
| Flood year | 1889 | Large ﬂood in the middle and lower reaches | 1491 | Liu et al. [[8](#_ENREF_8)] | 1308±280 |
|  | 1890 | Large ﬂood in the middle reach | 1102 |  |  |
|  | 1906 | Large ﬂood in the middle and lower reaches | 1097 |  |  |
|  | 1921 | Large ﬂood in the upper and middle reaches | 1315 |  |  |
|  | 1924 | Large ﬂood in the upper and middle reaches | 1136 |  |  |
|  | 1926 | Large ﬂood in the upper and middle reaches | 901 |  |  |
|  | 1931 | Extreme ﬂood in the whole reach | 1583 |  |  |
|  | 1935 | Extreme ﬂood in the middle reach | 1804 |  |  |
|  | 1949 | Large ﬂood in the middle and lower reaches | 1045 |  |  |
|  | 1954 | Extreme ﬂood in the whole reach | 1702 |  |  |
|  | 1969 | Large ﬂood in the middle and lower reaches | 1246 |  |  |
|  | 1980 | Large ﬂood in the middle and lower reaches | 1348 |  |  |
|  | 1983 | Large ﬂood in the middle reach | 1613 |  |  |
|  | 1991 | Large ﬂood in the middle and lower reaches | 825 |  |  |
|  | 1995 | Large ﬂood in the middle and lower reaches | 1268 |  |  |
|  | 1996 | Large ﬂood in the middle reach | 1420 |  |  |
|  | 1998 | Extreme ﬂood in the whole reach | 1260 |  |  |
|  | 1999 | Extreme ﬂood in the middle and lower reaches | 962 |  |  |
|  | 2016 | Large ﬂood in the middle and lower reaches | 1325 | Zhang et al. [[9](#_ENREF_9)] |  |
|  | 2020 | Large ﬂood in the middle and lower reaches | 1712 | Wei et al. [[10](#_ENREF_10)] |  |
| Drought year | 1900 | Extreme drought in the middle reaches | 646 | Li et al. [[5](#_ENREF_5)] | 812±117 |
|  | 1901 | Extreme drought in the middle reaches | 836 |  |  |
|  | 1902 | Extreme drought in the middle reaches | 644 |  |  |
|  | 1928 | Extreme drought in the middle reaches | 739 |  |  |
|  | 1966 | Extreme drought in the middle reaches | 796 |  |  |
|  | 1972 | Extreme drought in the middle reaches | 895 |  |  |
|  | 2001 | Extreme drought in the middle reaches | 853 | Jiang et al.[[11](#_ENREF_11)] |  |
|  | 2006 | Extreme drought in the upper and middle reaches | 941 | Dai et al. [[12](#_ENREF_12)] |  |
|  | 2022 | Extreme drought in whole reach | 959 | Ma et al. [[13](#_ENREF_13)] |  |

**2)** **Literature-reported paleo-flood events occurring in the MYV for the period ~4.0 kyr BP**

These past flood events were collated and plotted alongside HS4 δ^44/42^Ca-derived rainfall (mm/year) (Fig. 3). The age span of the flood layers was constrained by the ages of the top and bottom cultural layers. The C_combine function of OxCal V4.4 was used to combine the radiocarbon ages of cultural layers above/below the paleo-flood layer (Table S4), and produced a time interval for the occurrence of floods in MYV during ~4.0 kyr BP.

**Table S4.** Summary of the paleo-flood sediments records in the MYV during ~4.0 kyr BP, as displayed in Fig. 3e. The published radiocarbon data from Zhongqiao and Shijiahe sites characterised with complete raw radiocarbon data were calibrated using the R_date function of OxCal V4.4 and the IntCal20 calibration curve [[14](#_ENREF_14),[15](#_ENREF_15)]. No calibration processes for the published radiocarbon data from the Zhongba site were carried out due to the lack of raw radiocarbon data [[16](#_ENREF_16)].

| **Profile sites** | **Profile stratigraphy (from bottom to top)** | **Original published ^14^C** | | **Updated ^14^C** | | **References** |
| --- | --- | --- | --- | --- | --- | --- |
|  |  | **Age (yr BP)** | **±2s (yr)** | **Age (yr BP)** | **±2s (yr)** |  |
| Zhongqiao | Post-Shijiahe Culture | 3410 | 40 | 3410 | 44 | Wu et al. [[14](#_ENREF_14)] |
|  | Paleoflood |  |  |  |  |  |
|  | Post-Shijiahe Culture | 4168 | 81 | 4167 | 88 |  |
| Shijiahe | Paleoflood |  |  |  |  | Jia et al. [[15](#_ENREF_15)] |
|  | Post-Shijiahe Culture | 4156 | 92 | 4117 | 129 |  |
| Zhongba | Shang Dynasty | 3520 | 120 |  |  | Zhu et al. [[16](#_ENREF_16)] |
|  | Paleoflood |  |  |  |  |  |
|  | Post-Shijiahe Culture | 4055 | 35 |  |  |  |

**3) Radiocarbon dates of archaeological artefacts (displayed in main manuscript Fig. 3c)**

Summed Probability Distributions (SPD) of archaeological artefact ages are displayed in Fig. 3. The underlying data (of which 232 ages relate specifically to the MYV) is from an archaeological database spanning the Neolithic and Bronze Age (~8.0 to 2.0 kyr BP) [[17](#_ENREF_17)]. Of the 232 MYV archaeological dates, 32, 28 and 11 are associated with the Qujialing, Early-Middle Shijiahe and post-Shijiahe cultures respectively, with quality control details in [[17](#_ENREF_17)]. Archaeological dates were calibrated using the R_date function of OxCal V4.4 and the IntCal20 calibration curve [[3](#_ENREF_3)] (Fig. S7). Summed probability values were generated with the Sum function of OxCal V4.4. The calibrated data were standardised by X_i_/X_max_, where X_i_ is each value and X_max_ is the maximum value in the series. The SPDs were smoothed using a Savitzky–Golay filter with a 40-point (200 years) in the Origin software.


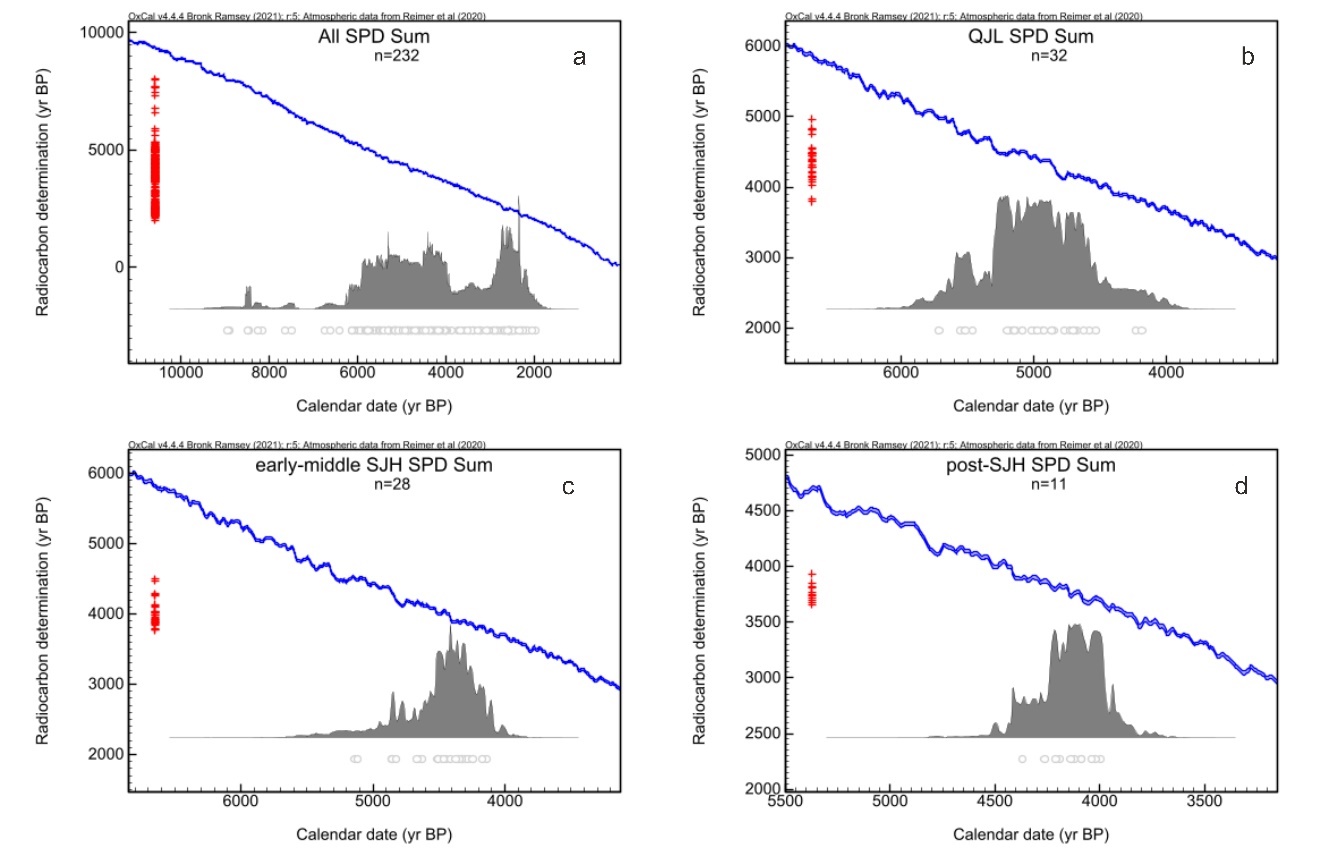


**Figure S7.** Sum of probability distribution (SPD) of archaeological radiocarbon dates. (a) All radiocarbon dates in the MYV. (b) Radiocarbon dates of Qujialing culture. (c) Radiocarbon dates of early-middle Shijiahe culture. (d) Radiocarbon dates of post-Shijiahe culture.

**4) Locations of paleoclimate records in East China (displayed in main manuscript Fig. 5)**


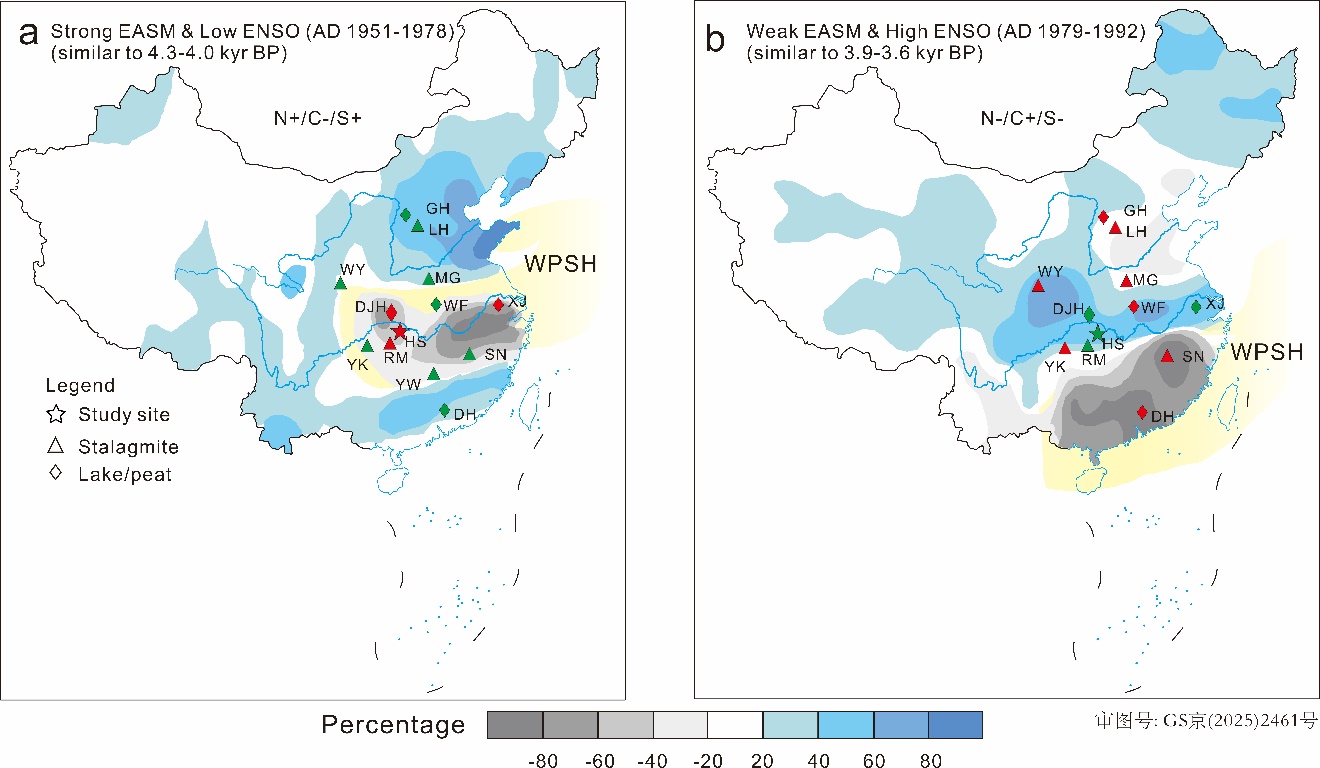


**Figure S8.** Location of climate proxy records displayed in our Fig. 5 plotted on top of modern climatology data adapted from Figs. 2 of [[18](#_ENREF_18),[19](#_ENREF_19)]. Blue colours (Grey colours) denote positive (negative) percentages from the mean summer rainfall amount. Green and red symbols (pentagram, triangles) represent wet and dry conditions, respectively. GH: Gonghai Lake [[20](#_ENREF_20)]; LH: Lianhua Cave [[21](#_ENREF_21)]; WY: Wuya Cave [[22](#_ENREF_22)]; MG: Magou Cave [[23](#_ENREF_23)]; WF: Wufan Lake [[24](#_ENREF_24)]; DJH: Dajiuhu Peat [[25](#_ENREF_25)]; HS: Heshang Cave (this study); RM: Remi Cave [[26](#_ENREF_26)]; YK: Yangkou Cave [[27](#_ENREF_27)]; YW: Yuwang Cave [[28](#_ENREF_28)]; SN: Shennong Cave [[29](#_ENREF_29)]; XJ: Xinjie Peat [[30](#_ENREF_30)]; DH: Dahu Peat [[31](#_ENREF_31)].

**5) Geomorphological setting in the Jianghan-Dongting floodplain**

The Jianghan-Dongting basin is situated in the Middle Yangtze Valley (MYV), encompassing the Jianghan basin on the north side of the river and the Dongting basin on the south side. Encircled by mountain ranges, this basin features a centrally positioned alluvial-lacustrine plain [[32](#_ENREF_32),[33](#_ENREF_33)]. The landform of the high alluvial plain (30–40 m asl) located at the edge of the basin, adjacent to the mountains, particularly on both banks of the Yangtze River in the western part of the basin, exhibits a flat topography primarily composed of Quaternary fluvial sediments [[32](#_ENREF_32),[34](#_ENREF_34)]. The alluvial-lacustrine plain (<30 m asl) constitutes the central region with relatively even terrain and Quaternary lacustrine deposits [[32](#_ENREF_32)]. Subtropical evergreen broadleaf forests dominate this area's vegetation type [[34](#_ENREF_34),[35](#_ENREF_35)]. The basin possesses well-developed drainage systems characterized by an abundance of rivers and lakes. The Yangtze River enters from the northwest into this basin while its largest tributary, the Hanshui River, flows southeast through the Jianghan Plain after entering from the north. In addition, there exists a hydrographic network comprising Dongting Lake in the southern part of this basin. Sedimentary and documentary records indicate that rivers within the MYV have undergone frequent course changes marked by frequent levee breaches, oxbow lake formation, and channel erosion events.

**6) Archaeological background information**

The Shijiahe main site is located in the northern Jianghan floodplain, situated north of the Hanshui River, and south of a piedmont zone with an elevation ranging from 30 to 50 m [[33](#_ENREF_33),[36](#_ENREF_36)]. The site has been surveyed and excavated since the 1950s. After several decades of survey and fieldwork, the current consensus is that Shijiahe consists of a core hub about 1.8 km^2^ in size that is surrounded by an earthen wall and a moat [[36](#_ENREF_36)]. Probing surveys and excavations in several areas beyond the core hub have revealed Late Neolithic remains at more than forty localities with an 8.0 km^2^ area [[36](#_ENREF_36)]. Ancient human occupation began in the core hub of Shijiahe as early as ~5.9 kyr BP, but the major periods of occupation within it are the Upper Qujialing (~5.3 to 4.5 kyr BP) and Shijiahe (~4.6 to 3.9 kyr BP) [[36](#_ENREF_36),[37](#_ENREF_37)]. The Qujialing culture thrived and spread throughout the MYV, witnessing a period of cultural prosperity characterized by numerous prehistoric settlements and urban sites [[36-39](#_ENREF_36)]. Rice cultivation underwent a significant expansion to sustain the growing population at this period [[40-42](#_ENREF_40)]. During the Shijiahe period, settlements exhibited a hierarchical structure, reaching their peak in terms of number during the Neolithic era [[36](#_ENREF_36)]. The ancient city site of Shijiahe emerged as the capital of the MYV, indicating that prehistoric civilization in this region had entered its early stage [[36](#_ENREF_36)]. The layout of the city, reconstructed through archaeological excavations, reveals a well-designed urban structure comprising sacrificial palaces, dwellings, walls, and artificial ditches [[36](#_ENREF_36)] (Fig. S9a). The ancient city ruins of Tanjialing are considered one of China's largest prehistoric cities, while the Yinxintai site is regarded as the largest prehistoric ritual site in the MYV (Fig. S9a). Excavations at Tanjialing unearthed jadewares with manufacturing technologies representing a pinnacle level in prehistoric China and even East Asia [[36](#_ENREF_36)] (Fig. S9b). However, around 3.9 kyr BP this flourishing culture collapsed with numerous settlements being abandoned and a dramatic decline in sites observed. This decrease persisted until approximately 3.55 kyr BP, during which human activities were revived again in the MYV during the Shang Dynasty [[32](#_ENREF_32)].

​
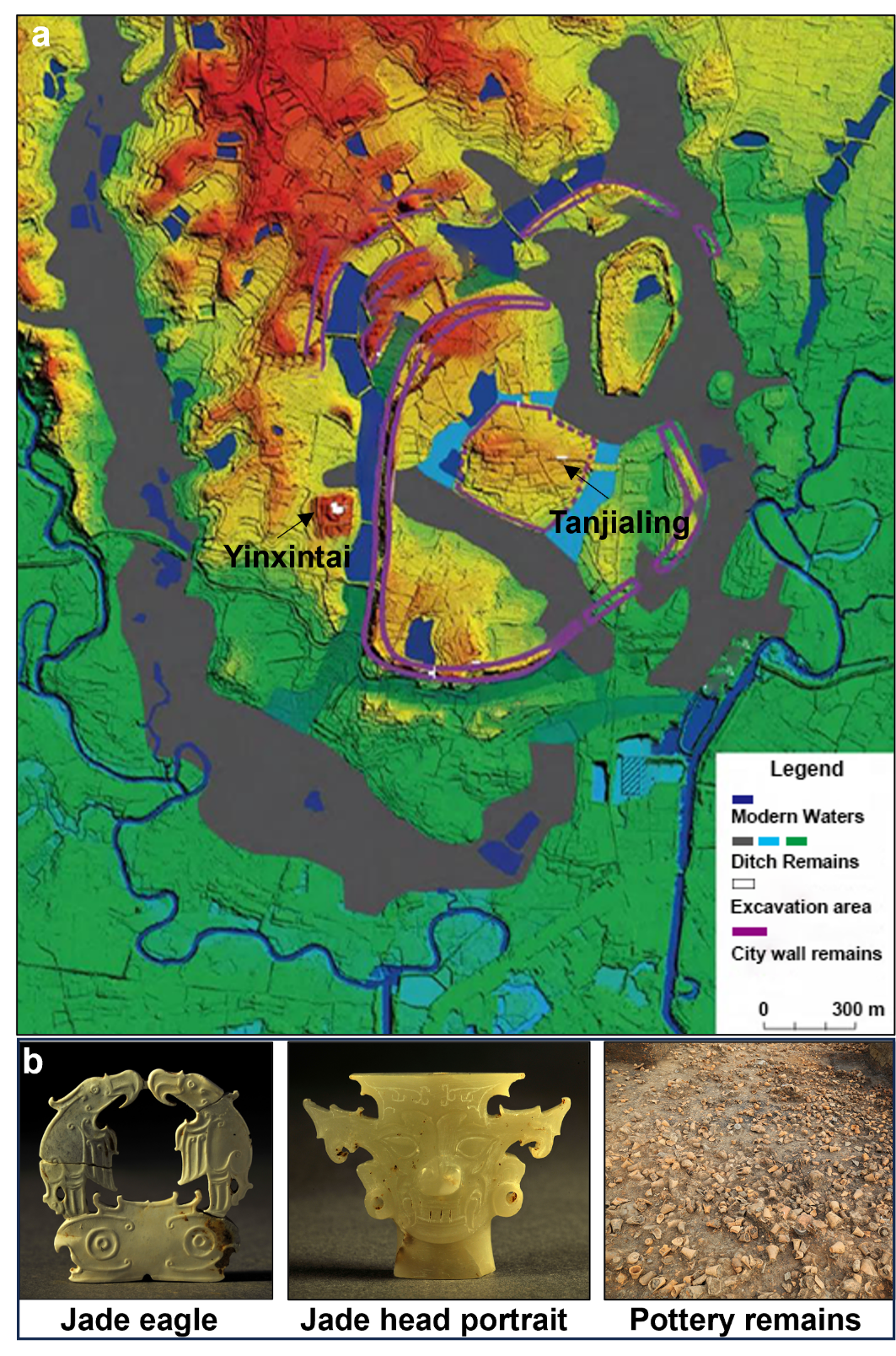
​

**Figure S9.** Shijiahe ancient city. (a) Digital elevation map of the Shijiahe ancient city showing the ditches and city wall constructed by the Shijiahe people. (b) Handmade jade wares and pottery excavated in the Shijiahe ancient city. (Photo credit: <https://archaeology.pku.edu.cn/info/1030/3480.htm>, <http://en.hubei.gov.cn/news/newslist/201701/t20170111_938425.shtml>)

Dataset S1 (separate file). Dataset S1_Stalagmite U/Th ages and layer countings.

Dataset S2 (separate file). Dataset S2_Archaeological radiocarbon dates.

Dataset S3 (separate file). Dataset S3_HS4 geochemistry data.

**REFERENCES**

1. Cheng H, Edwards R, Shen C-C *et al*. Improvements in ^230^Th dating, ^230^Th and ^234^U half-life values, and U–Th isotopic measurements by multi-collector inductively coupled plasma mass spectrometry. *Earth Planet Sci Lett* 2013; **s 371–372**: 82–91.

2. Hu C, Henderson GM, Huang J *et al*. Quantification of Holocene Asian monsoon rainfall from spatially separated cave records. *Earth Planet Sci Lett* 2008; **266**: 221-32.

3. Ramsey CB. Deposition models for chronological records. *Quat Sci Rev* 2008; **27**: 42-60.

4. Owen RA, Day CC, Hu CY *et al*. Calcium isotopes in caves as a proxy for aridity: Modern calibration and application to the 8.2 kyr event. *Earth Planet Sci Lett* 2016; **443**: 129-38.

5. Li X, Cui X, He D *et al*. Evaluation of the Heshang Cave stalagmite calcium isotope composition as a paleohydrologic proxy by comparison with the instrumental precipitation record. *Sci Rep* 2018; **8**: 2615.

6. Owen RA, Day CC, Henderson GM. CaveCalc: A new model for speleothem chemistry & isotopes. *Comput Geosci* 2018; **119**: 115-22.

7. Day CC, Henderson GM. Controls on trace-element partitioning in cave-analogue calcite. *Geochim Cosmochim Acta* 2013; **120**: 612-27.

8. Liu XJ, Min FY, Kettner AJ. The impact of large to extreme flood events on floodplain evolution of the middle and lower reaches of the Yangtze River, China. *CATENA* 2019; **176**: 394-409.

9. Zhang W, Jin F-F, Stuecker MF *et al*. Unraveling El Niño's impact on the East Asian Monsoon and Yangtze River summer flooding. *Geophys Res Lett* 2016; **43**: 11,375-82.

10. Wei K, Ouyang C, Duan H *et al*. Reflections on the Catastrophic 2020 Yangtze River Basin Flooding in Southern China. *Innovation (Camb)* 2020; **1**: 100038.

11. Jiang X, Li Y, Wang X. Water vapor transport over China and its relationship with drought and flood in Yangtze River Basin. *J Geogr Sci* 2009; **19**: 153-63.

12. Dai Z, Chu A, Stive M *et al*. Unusual salinity conditions in the Yangtze estuary in 2006: impacts of an extreme drought or of the Three Gorges Dam? *Ambio* 2011; **40**: 496-505.

13. Ma M, Qu Y, Lyu J *et al*. The 2022 extreme drought in the Yangtze River Basin: Characteristics, causes and response strategies. *River* 2022; **1**: 162-71.

14. Wu L, Zhu C, Ma C *et al*. Mid-Holocene palaeoflood events recorded at the Zhongqiao Neolithic cultural site in the Jianghan Plain, middle Yangtze River Valley, China. *Quat Sci Rev* 2017; **173**: 145-60.

15. Jia M, Li C, Mao X *et al*. Climate–human–environment interactions in the middle Yangtze Basin (central China) during the middle Holocene, based on pollen and geochemical records from the Sanfangwan Site. *CATENA* 2021; **204**: 105357.

16. Zhu C, Zheng C, Ma C *et al*. Identifying paleoflood deposits archived in Zhongba Site, the Three Gorges reservoir region of the Yangtze River, China. *Chin Sci Bull* 2005; **50**: 2493-504.

17. He K, Lu H, Jin G *et al*. Antipodal pattern of millet and rice demography in response to 4.2 ka climate event in China. *Quat Sci Rev* 2022; **295**: 107786.

18. Ding Y, Wang Z, Sun Y. Inter-decadal variation of the summer precipitation in East China and its association with decreasing Asian summer monsoon. Part I: Observed evidences. *Int J Climatol* 2008; **28**: 1139-1161.

19. Rao Z, Li Y, Zhang J *et al*. Investigating the long-term palaeoclimatic controls on the δD and δ^18^O of precipitation during the Holocene in the Indian and East Asian monsoonal regions. *Earth Sci Rev* 2016; **159**: 292-305.

20. Chen F, Xu Q, Chen J *et al*. East Asian summer monsoon precipitation variability since the last deglaciation. *Sci Rep* 2015; **5**: 11186.

21. Dong J, Shen CC, Kong X *et al*. Reconciliation of hydroclimate sequences from the Chinese Loess Plateau and low-latitude East Asian Summer Monsoon regions over the past 14,500 years. *Palaeogeogr Palaeoclimatol Palaeoecol* 2015; **435**: 127-135.

22. Tan L, Shen C-C, Cai Y *et al*. Great flood in the middle-lower Yellow River reaches at 4000 a BP inferred from accurately-dated stalagmite records. *Science Bulletin* 2018; **63**: 206-208.

23. Cai Y, Cheng X, Ma L *et al*. Holocene variability of East Asian summer monsoon as viewed from the speleothem δ18O records in central China. *Earth Planet Sci Lett* 2021; **558**: 116758.

24. Jiang S, Luo W, Zhou X *et al*. Collapse of prehistoric cultures in central-eastern China linked to the El Niño-like states during the 4.2 ka event. *Global Planet Change* 2025; **248**: 104772.

25. Liu H, Gu Y, Yu Z *et al*. Holocence peatland water regulation response to ∼1000-year solar cycle indicated by phytoliths in central China. *J Hydrol* 2020; **589**: 125169.

26. Wang T, Li D, Cheng X *et al*. Hydroclimatic changes in south-central China during the 4.2 ka event and their potential impacts on the development of Neolithic culture. *Quat Res* 2022; **615**: 1-14.

27. Chen C, Wu Y, Li J *et al*. Hydrological changes in the East Asian monsoon region around 4.2 ka precisely reconstructed from multi-proxy stalagmites. *Quat Sci Rev* 2025; **357**: 109321.

28. Yin J, Wang Z, Wu X *et al*. Hydroclimate change during the transition of mid-to late Holocene and its potential impacts on late Neolithic settlements in middle Yangtze Basin, Central-South China. *Global Planet Change* 2025; **251**: 104834.

29. Zhang H, Cheng H, Sinha A *et al*. Collapse of the Liangzhu and other Neolithic cultures in the lower Yangtze region in response to climate change. *Sci Adv* 2021; **7**: eabi9275.

30. Lu F, Ma C, Zhu C *et al*. Variability of East Asian summer monsoon precipitation during the Holocene and possible forcing mechanisms. *Clim Dyn* 2019; **52**: 969-989.

31. Zhou W, Yu X, Jull AJT *et al*. High-resolution evidence from southern China of an early Holocene optimum and a mid-Holocene dry event during the past 18,000 years. *Quat Res* 2004; **62**: 39-48.

32. Zhao C, Mo D. Holocene hydro-environmental evolution and its impacts on human occupation in Jianghan-Dongting Basin, middle reaches of the Yangtze River, China. *J Geogr Sci* 2020; **30**: 423-38.

33. Xie S, Evershed RP, Huang X *et al*. Concordant monsoon-driven postglacial hydrological changes in peat and stalagmite records and their impacts on prehistoric cultures in central China. *Geology* 2013; **41**: 827-30.

34. Wu L, Lu S, Zhu C *et al*. Holocene Environmental Archaeology of the Yangtze River Valley in China: A Review. *Land* 2021; **10**: 302.

35. Li B, Zhu C, Wu L *et al*. Relationship between environmental change and human activities in the period of the Shijiahe culture, Tanjialing site, Jianghan Plain, China. *Quat Int* 2013; **308-309**: 45-52.

36. Li T, Underhill AP, Shan S. Shijiahe and its implications for understanding the development of urbanism in Late Neolithic China. *J Urban Archaeol* 2023; **7**: 31-49.

37. Zhang C. The Qujialing–Shijiahe Culture in the Middle Yangzi River Valley. In: Underhill AP, ed. *A Companion to Chinese Archaeology*. Wiley-Blackwell; 2013:510-34.

38. Li B, Liu H, Wu L *et al*. Linking the vicissitude of Neolithic cities with mid Holocene environment and climate changes in the middle Yangtze River, China. *Quat Int* 2014; **321**: 22-8.

39. Liu L, Chen X. *The archaeology of China: from the late Paleolithic to the early Bronze Age*. Cambridge University Press; 2012.

40. Fuller DQ, Qin L, Zheng Y *et al*. The Domestication Process and Domestication Rate in Rice: Spikelet Bases from the Lower Yangtze. *Science* 2009; **323**: 1607-10.

41. Gutaker RM, Groen SC, Bellis ES *et al*. Genomic history and ecology of the geographic spread of rice. *Nat Plants* 2020; **6**: 492-502.

42. Li L, Wu L, Zhu C *et al*. Relationship between archaeological sites distribution and environment from 1.15 Ma BP to 278 BC in Hubei Province. *J Geogr Sci* 2011; **21**: 909-25.
